# Climate change shifts almond bloom dates in Afghanistan

**DOI:** 10.1007/s00484-026-03288-0

**Published:** 2026-07-28

**Authors:** Atifullah Shinwari, Lars Caspersen, Katja Schiffers, Eike Luedeling

**Affiliations:** Horticultural Sciences, Institute of Crop Science and Resource Conservation (INRES), University of Bonn, 53121 Bonn, Germany

**Keywords:** Almond phenology, Climate change, Bloom projection, PhenoFlex framework, Flowering shift

## Abstract

Climate change is reshaping the spring phenology of temperate fruit and nut trees worldwide, causing uneven bud break and yield losses across major production regions. Almond trees, including those cultivated in Afghanistan, are susceptible to these shifts. Afghanistan produces over 50 almond varieties, with official exports worth around USD 40 million in 2024. Yet, this value remains below potential due to multiple challenges along the value chain, one of which is the ongoing temperature increase affecting phenology and yields.

We projected the flowering dynamics of 51 Afghan almond cultivars using long-term temperature data (1980–2023) and phenological observations (2011–2023). Two historical reference periods (1980 and 2020) and future projections for 2050 and 2085 were simulated under three Shared Socioeconomic Pathway (SSP) scenarios (SSP1-2.6, SSP2-4.5, SSP5-8.5) based on 15 General Circulation Models. Bloom predictions were obtained using the PhenoFlex modeling framework with a recently developed combined-fitting approach. The model accurately reproduced observed bloom patterns, achieving a Root Mean Square Error of Prediction below four days for all cultivars. Compared to 1980, bloom dates advanced by roughly two weeks by 2020. Future projections suggest additional advances of up to 16 days for most cultivars, whereas a few exhibited delays of up to 12 days. Pronounced shifts exceeding seven days were found in about 25% of all predictions driven by specific cultivars, especially by 2085 under intermediate and high-emission scenarios. These results emphasize the significant and ongoing changes in almond phenology, highlighting the importance of adaptive management to sustain production and export potential under future climates.

**Supplementary Information:**

The online version contains supplementary material available at 10.1007/s00484-026-03288-0.

## Introduction

Almond (*Prunus dulcis* (Mill.) D.A. Webb) production in Afghanistan plays a crucial role in the country’s agricultural economy. In 2009, the FAO ranked Afghanistan as the ninth-largest almond producer globally. The country produces both sweet and bitter almond varieties (ANHDO 2017), which are primarily grown in the northern provinces of Samangan, Balkh and Kunduz, as well as in the southern provinces Uruzgan, Zabul and Daikundi. According to official estimates, almonds covered a total area of approximately 37,000 hectares with an estimated production of 67,000 metric tons in the year 2024 (NSIA 2025). Afghanistan is home to a rich diversity of almond germplasm, with more than 50 varieties exhibiting distinct morphological and genetic traits (Giordani et al. 2014, 2017).

In recent decades, declining winter chill accumulation has affected tree fruit and nut production in various regions worldwide (Benmoussa et al. 2020; Fernandez et al. 2021). In Afghanistan, projections indicate a significant reduction in winter chill, particularly in lowland areas (Shinwari et al. 2025). A localized assessment also reported low chill conditions in a subtropical province (Nangarhar) in eastern Afghanistan (Finetto 2014). For pessimistic scenarios, future projections suggest an increase in minimum and maximum temperatures by up to 2.5 °C between 2020 and 2059 and up to 5 °C between 2060 and 2099 (Sediqi et al. 2022). Such warming trends emphasize the need to investigate how climate change will influence phenological events in temperate fruit trees, including almonds.

Like other deciduous fruit and nut trees, almonds undergo a period of dormancy after shedding their leaves in autumn to withstand the cold season (Campoy et al. 2011). Dormancy release requires sequential exposure to cold and warm temperatures, a process divided into two phases: (1) endodormancy, during which meristematic growth is temporarily suspended even under favorable environmental conditions (chilling phase), and (2) ecodormancy, when tree buds resume growth in response to rising temperatures in spring (forcing phase) (Fadón et al. 2020).

Rising temperatures impact spring phenology in different ways. Inadequate exposure to chill due to warming compromises dormancy release and affects the subsequent spring phases (Rohde and Bhalerao 2007; Fadón et al. 2020). This may lead to shifts in bloom patterns, such as advances or delays, depending on cultivars and on which specific dormancy phases experience rising temperatures (Luedeling and Gassner 2012). Spring phenology is generally advanced when temperatures in spring increase, whereas higher temperatures in autumn and winter may delay bloom (Guo et al. 2015). This has been observed in Sfax, Tunisia, where most almond cultivars showed delayed bloom in response to high temperatures during the chilling phase (Benmoussa et al. 2017). Temperature-driven changes in bloom timing may also affect the interval between flowering and leaf emergence, potentially increasing the risk of frost damage, pest outbreaks, prolonged growing seasons, and carbohydrate allocation mismatches (Guo et al. 2023). Cultivar-specific phenological variation is crucial to consider. A previous study on the correlation between the reproductive phenology calendar and temperature showed variation among Mediterranean almond cultivars, including differences in flowering phenology, duration, and agroclimatic requirements such as chill and heat needs, highlighting the importance of environmental differences between cold and warm regions (Sakar et al. 2023).

Afghanistan’s national almond collection plays a critical role in preserving the genetic diversity of this species. Since 2011, the collection has maintained detailed characterizations of almond cultivars, and it collects phenological records, including bloom dates for different cultivars (Giordani et al. 2014, 2017). Despite significant advances in phenology modeling, uncertainty remains about how various cultivars will respond to future climate conditions, particularly in regions lacking extensive long-term phenology data. Chill shortfalls and rising temperatures due to climate change are already evident (Sediqi et al. 2022; Shinwari et al. 2025), yet studies on phenology trends and bloom shifts remain scarce in Afghanistan. These records provide a valuable foundation for modeling future phenology trends and for estimating winter chill requirements. In this study, we use the PhenoFlex modeling framework to project the bloom dates of almond cultivars in Kunduz province under future climate scenarios and to assess the cultivar-specific chill and heat requirements that influence these responses. Instead of using the standard PhenoFlex model, we leverage recent advancements in model calibration (Caspersen et al. 2026), which allow estimating shared and cultivar-specific parameters jointly. This approach allows us to utilize large multi-cultivar datasets more effectively; therefore, we apply it across many cultivars to gain insights into cultivar variability. Our findings aim to help growers and stakeholders anticipate potential risks that could impact Afghanistan’s almond industry and its associated value chain.

## Materials and methods

### Study area

**Fig. 1:**
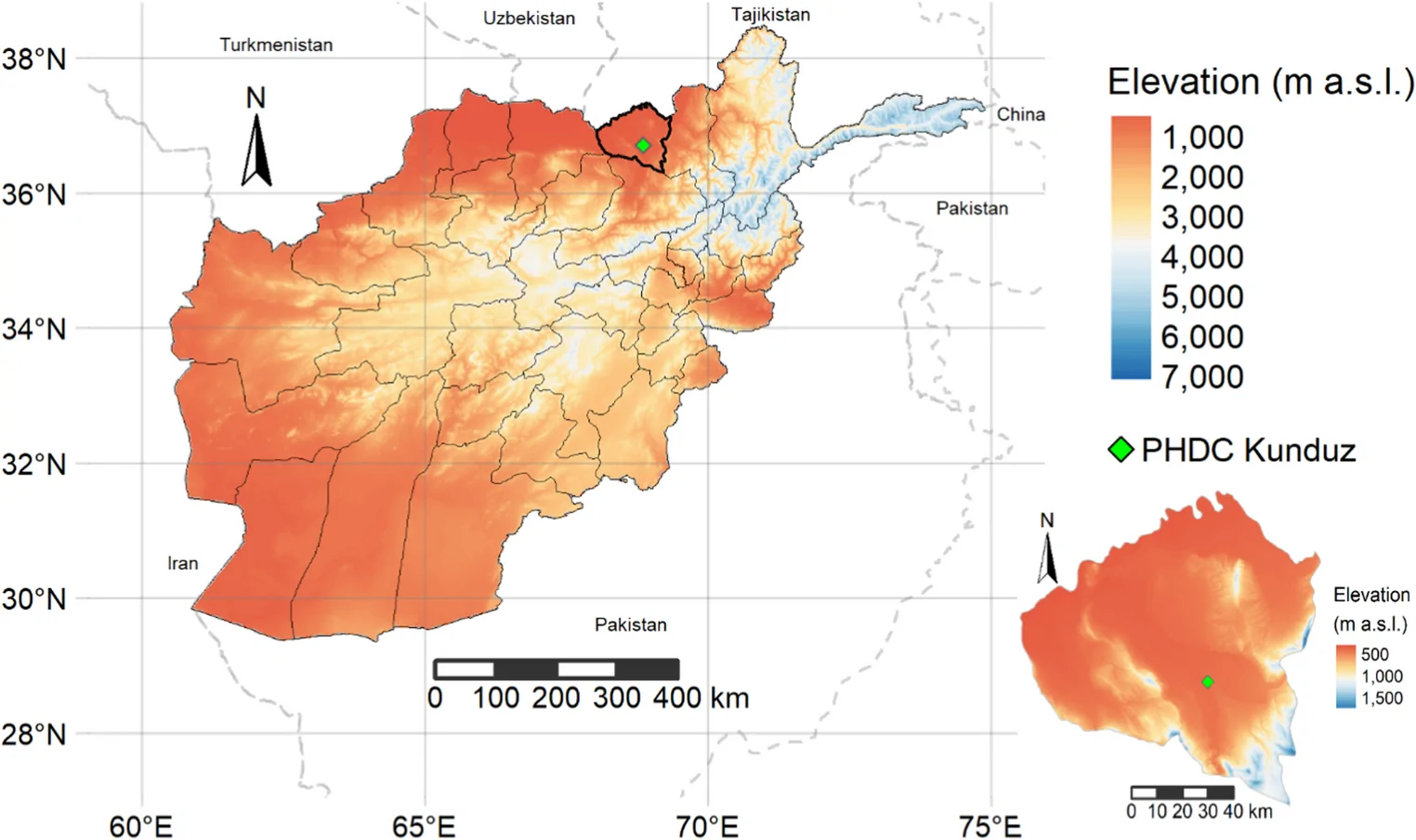
Elevation map of Afghanistan showing the location of the Perennial Horticulture Development Centre (PHDC) in Kunduz province, where the almond phenology data used in this study were collected

In this study, we investigated the flowering phenology of almond varieties grown in northern Afghanistan. The almond varieties were collected from various almond-producing regions across the country and cultivated at the Perennial Horticulture Development Centre (PHDC) in Kunduz and Balkh provinces between 2007 and 2010 (Giordani et al. 2014, 2017). These orchards, officially known as the National Collection of Varieties of Fruits and Nuts of Afghanistan—Almonds, are located at the Kunduz research farm (36.70893° N latitude, 68.86145° E longitude) (Giordani et al. 2014, 2017) at an elevation of approximately 400 m above mean sea level (Fig. 1). The region features a continental climate, characterized by cold winters and hot, dry summers. Daily temperatures ranged from below zero in winter to as high as 40 °C during the peak summer months, according to meteorological data from the “Chahar Dara” weather station.

### Flowering phenology and temperature data

For the phenology dataset, we collected bloom dates of 51 almond cultivars from the experimental almond orchards at the PHDC in Kunduz, maintained by the Directorate of Agriculture, Irrigation and Livestock. The dataset spans 12 years, covering the period between 2011 and 2023. Data from 2013 were excluded from the analysis due to noticeable discrepancies that may have resulted from errors in data entry and compilation. We used ‘first bloom’ data, defined as the stage when 10% of flowers have opened. This bloom stage marks the onset of flowering and corresponds to BBCH stage 61 (Meier et al. 2009; Sakar et al. 2019).

For the same period, we obtained daily minimum and maximum temperature records from the “Chahar Dara” hydrometeorological station, located approximately 5 km away. This station, operated by the Directorate of Hydrometeorology of the Ministry of Energy and Water (MEW) of Afghanistan, has been recording standardized observations every 15 min with high reliability since 2008. For further analysis, we converted the daily records into hourly temperature data using the *chillR* package (version 0.76; Luedeling et al. 2024) in the R software environment (version 4.2.2; R Core Team 2022). For details, see the documentation of the ‘stack_hourly_temps’ function from the *chillR *package.

### PhenoFlex framework

We used the PhenoFlex model (Luedeling et al. 2021) to predict bloom dates for 51 almond varieties cultivated at the Kunduz PHDC. Based on the theoretical principles of the Dynamic Model (Fishman et al. 1987a, b) and the Growing Degree Hours (GDH) Model (Anderson et al. 1986), the PhenoFlex framework simulates dormancy progression and bud break, driven by hourly temperature data. Unlike other phenology models, PhenoFlex features a flexible transition between endodormancy and ecodormancy, allowing it to be fitted to observation data (Luedeling et al. 2021). The model has shown strong predictive performance and has been successfully applied to predict the phenology of temperate fruit trees, including almonds (Caspersen et al. 2026). Overall, the model contains 12 parameters.

### Model calibration and validation

We applied a recent advancement in the PhenoFlex calibration framework, published as the combined-fitting approach (Caspersen et al. 2026). Unlike standard PhenoFlex, which fits the model independently for each cultivar, this approach classifies the 12 model parameters into two categories: shared parameters, whose values are common across all cultivars, and cultivar-specific parameters. Among the nine shared parameters, six (*A*_*0*_, *A*_*1*_, *E*_*0*_, *E*_*1*_, *T*_*f*_, *slope*) originate from the Dynamic Model (Fishman et al. 1987a, b) and three (*T*_*b*_, *T*_*u*_, *T*_*c*_) from the Growing Degree Hours (GDH) Model (Anderson et al. 1986). Considering these parameters common to all varieties assumes that all almond varieties share a fundamental phenological response to chill and heat accumulation, allowing these parameters to be calibrated collectively and enhancing model calibration by leveraging a larger dataset that combines observations from multiple cultivars. The cultivar-specific parameters—chill requirement (*y*_*c*_), heat requirement (*z*_*c*_), and the transition between chill and heat accumulation (*s*_*1*_)—originate from the PhenoFlex Model (Luedeling et al. 2021) and are fitted individually for each variety.

To ensure model reliability, we employed a cross-validation procedure. We generated a season list from the hourly temperature data and randomly split the bloom data for each cultivar into calibration (75%) and validation (25%) subsets. These random splits were used to avoid overfitting and sensitivity to a single split, and to ensure reliable estimates of the bloom predictions. To estimate the model parameters for all varieties, we applied a global optimization method designed to minimize overall error. We chose an Enhanced Scatter Search with local refinement using Dynamic Hill Climbing, as implemented in the MEIGOR package (Egea et al. 2014). We also substituted parameters (*E*_*0*_, *E*_*1*_, *A*_*0*_, *A*_*1*_) and introduced intermediate parameters (θ*, *θ*_*c*_, *τ*, *π*_*c*_) to facilitate the calibration by expressing values in measurable units, thereby enhancing their interpretability and reducing the search space during optimization (Egea et al. 2020). The parameters θ* and *T*_*c*_ were kept contant throughout the analysis as they showed no significant impact on model performance. To further enhance robustness, we used a 10-fold cross-validation scheme for each cultivar. In each fold, we supplied a random set of calibration and validation bloom data to obtain predictions for each dataset. Model performance was evaluated by calculating the Root Mean Square Error of Prediction (RMSEP) and the Ratio of Performance to Inter-Quartile distance (RPIQ).

### Historical and future bloom prediction

To predict potential changes in the bloom dates of almond cultivars, we applied the calibrated models to both historical and future climate scenarios. For historical simulations, we obtained daily temperature records from 1980 to 2007 from the ERA5 dataset via ClimateEngine with a spatial resolution of 0.1 °C (~ 9.6 km). As a cloud-based platform, ClimateEngine offers access to remote sensing and climate model datasets through its publicly available app (Huntington et al. 2017). To correct for inherent biases, we applied Quantile Delta Mapping (QDM; Cannon et al. 2015) using observed temperature records from 2008 to 2023 for calibration and then merged the bias-adjusted data for 1980 to 2007 with the observed temperature records from 2008 to 2023. This allowed us to generate two historical reference scenarios, 1980 and 2020, to investigate changes in bloom dates over time. To calculate the typical conditions in the 1980 and 2020 scenario years, we applied running mean functions to the daily minimum and maximum temperatures for each calendar month across the complete weather. The running means were calculated over a 31-year window centered on each scenario year to include 15 years before and after the scenario year. The resulting values represented the typical conditions of the scenario years.

For future projections, we used the Shared Socioeconomic Pathway (SSP) scenarios SSP1-2.6, SSP2-4.5, and SSP5-8.5 from the Coupled Model Intercomparison Project Phase 6 (CMIP6; Eyring et al. 2016), retrieved from the Copernicus Climate Data Store (Copernicus Climate Change Service, Climate Data Store, 2021). These scenarios were obtained for 15 General Circulation Models (GCMs) and two future time periods, 2035–2065 (referred to as 2050) and 2070–2100 (referred to as 2085).

Using the RMAWGEN weather generator (Cordano and Eccel 2016), we produced 100 random realizations for all historical and future scenarios. Bloom dates were predicted by applying the previously calibrated models to the distributions of historical and future scenarios, GCMs, and time points. In addition, we estimated the percentage of years where the thermal needs are not fulfilled, which may result in bloom failure. For further analysis and visualization, we aggregated the projections from the 10 models using a weighted-mean approach, with the Ratio of Performance to Inter-Quartile distance (RPIQ) of the validation dataset as the weighting factor.

### Data analysis and visualization

The R software environment (version 4.2.2; R Core Team 2022) and in particular the *chillR* package (version 0.76; Luedeling et al. 2024) were used to implement all steps involved in this study.

## Results

### Performance of the combined-fitting approach

**Fig. 2:**
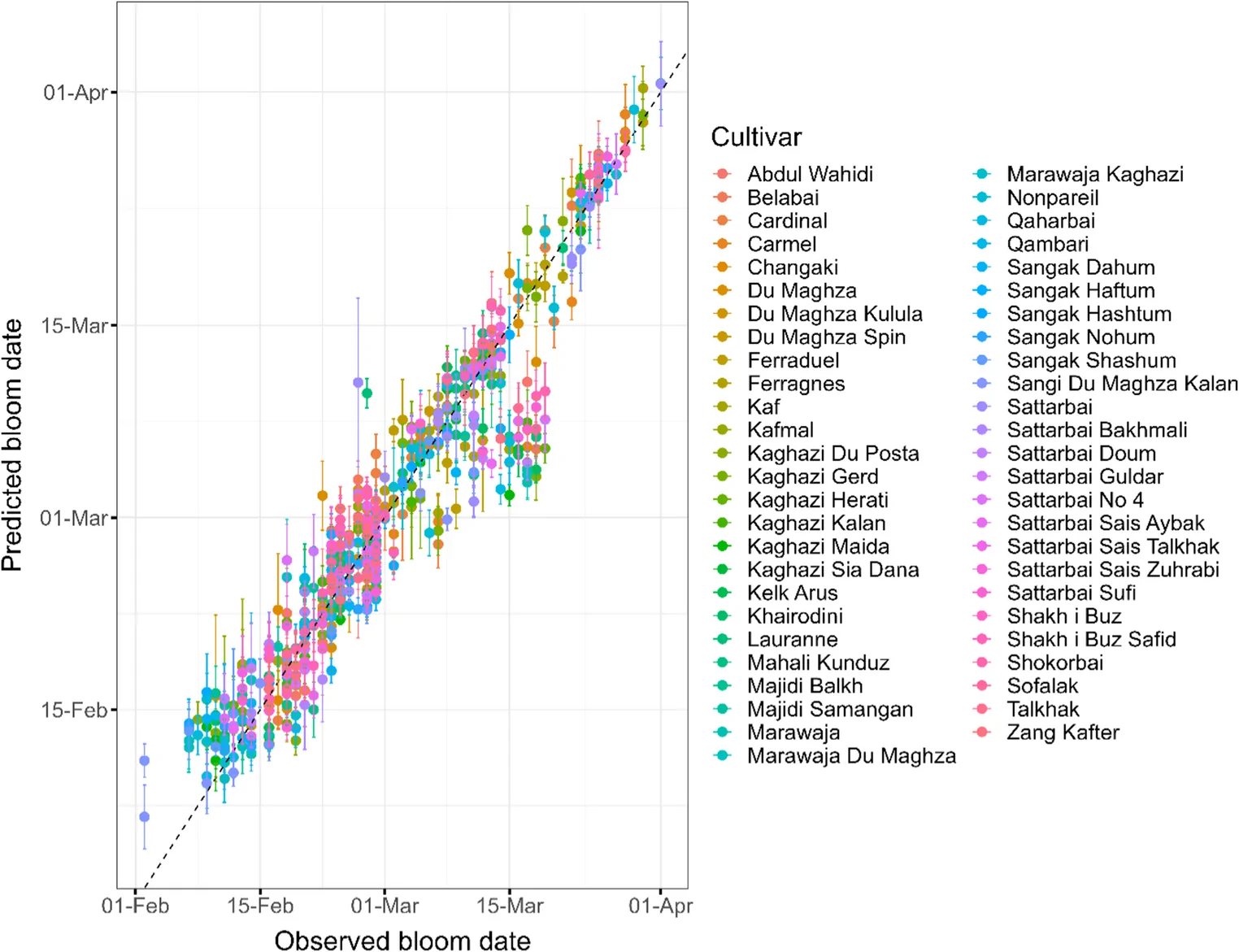
Weighted means of predicted bloom dates for 51 almond cultivars plotted over observed bloom dates, spanning 12 years, from 2011 to 2023 (excluding 2013). Error bars indicate the degree of variation (standard deviation) in predicted bloom dates across 10 cross-validation folds

The validation of the calibrated models showed a generally high performance (Fig. 2). For most almond cultivars, the predicted bloom dates closely corresponded to the observed dates. The Root Mean Square Error of Prediction (RMSEP) for all almond cultivars was consistently < 4 days, indicating a strong predictive capability of the model. Additionally, the Ratio of Performance to Inter-Quartile distance (RPIQ) averaged approximately 5, with a range from 3 to 7 (Fig. S1, S2 Supplementary Information), which indicates that the model was able to account for most of the observed variation in bloom dates. While most predictions were accurate, a few discrepancies were noted, particularly for 2014, where the model tended to predict bloom dates that were earlier than the observed ones. Upon further analysis, we identified an extreme cold spell in February 2014, which might have contributed to the model’s early-prediction, although the precise cause of this anomaly remains unclear (Fig. S3, Supplementary Information).

### Predicted bloom dates in historical scenarios

**Fig. 3:**
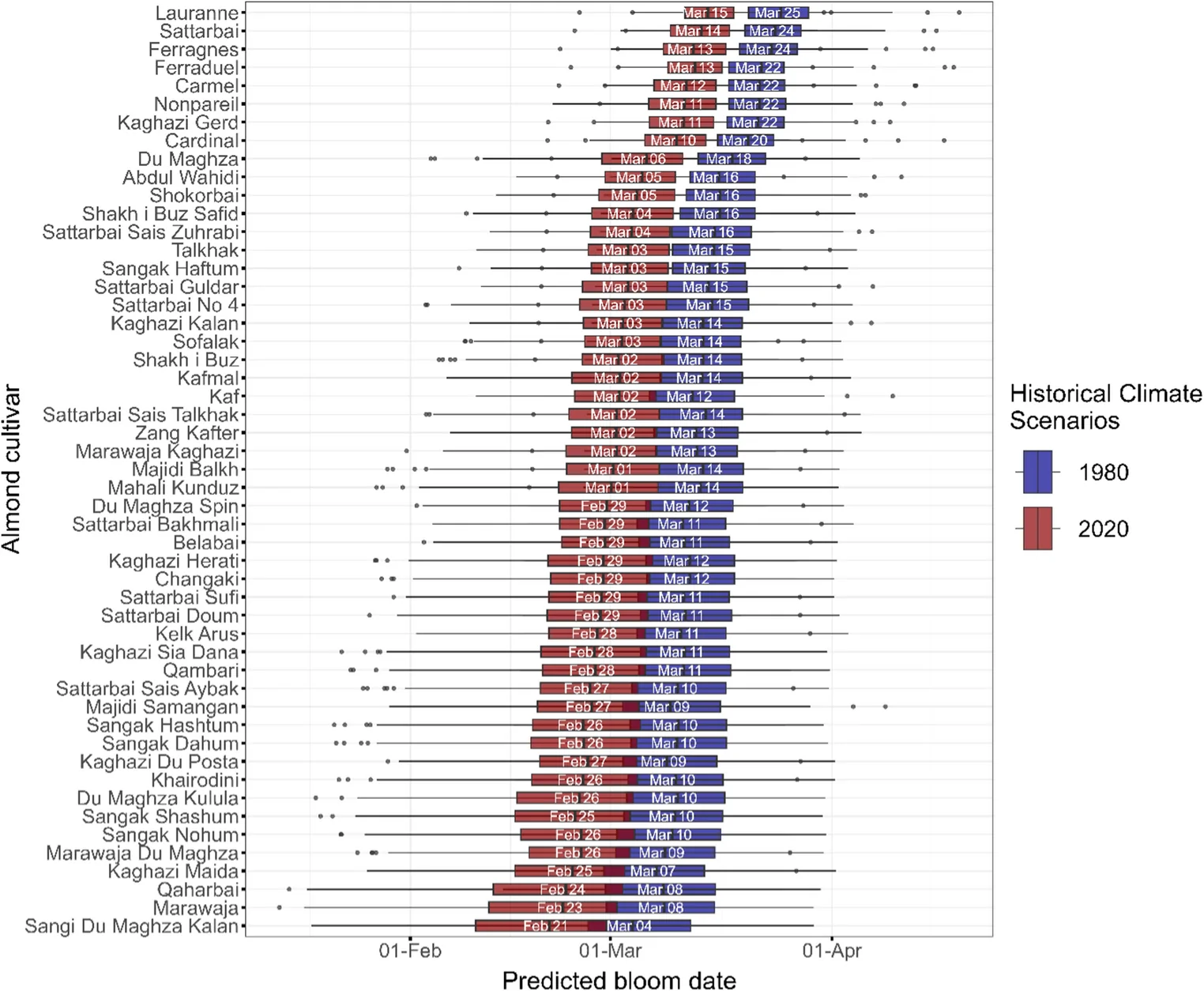
Predicted bloom dates for 51 almond cultivars under the historical climate scenarios representing conditions in 1980 (blue) and 2020 (red). Boxplots show distributions of predicted bloom dates based on 100 synthetic temperature scenarios, and the dates mentioned inside the boxes are calculated as their medians. Bloom dates were estimated with the PhenoFlex framework, with parameters calculated as weighted averages across 10 cross-validation folds of model calibration

We observed considerable variation in the predicted bloom dates between the historical scenarios representing conditions in 1980 and 2020 (Fig. 3). Across all almond cultivars, the median flowering date, derived from 100 random weather realizations, generally occurred approximately 10 to 15 days earlier in the 2020 scenario compared to 1980. A clear distinction emerged between early- and late-flowering cultivars, with ‘Sangi Du Maghza Kalan’ identified as the earliest-flowering variety and ‘Lauranne’ as the latest (Fig. 3).

### Predicted bloom dates in future scenarios

**Fig. 4:**
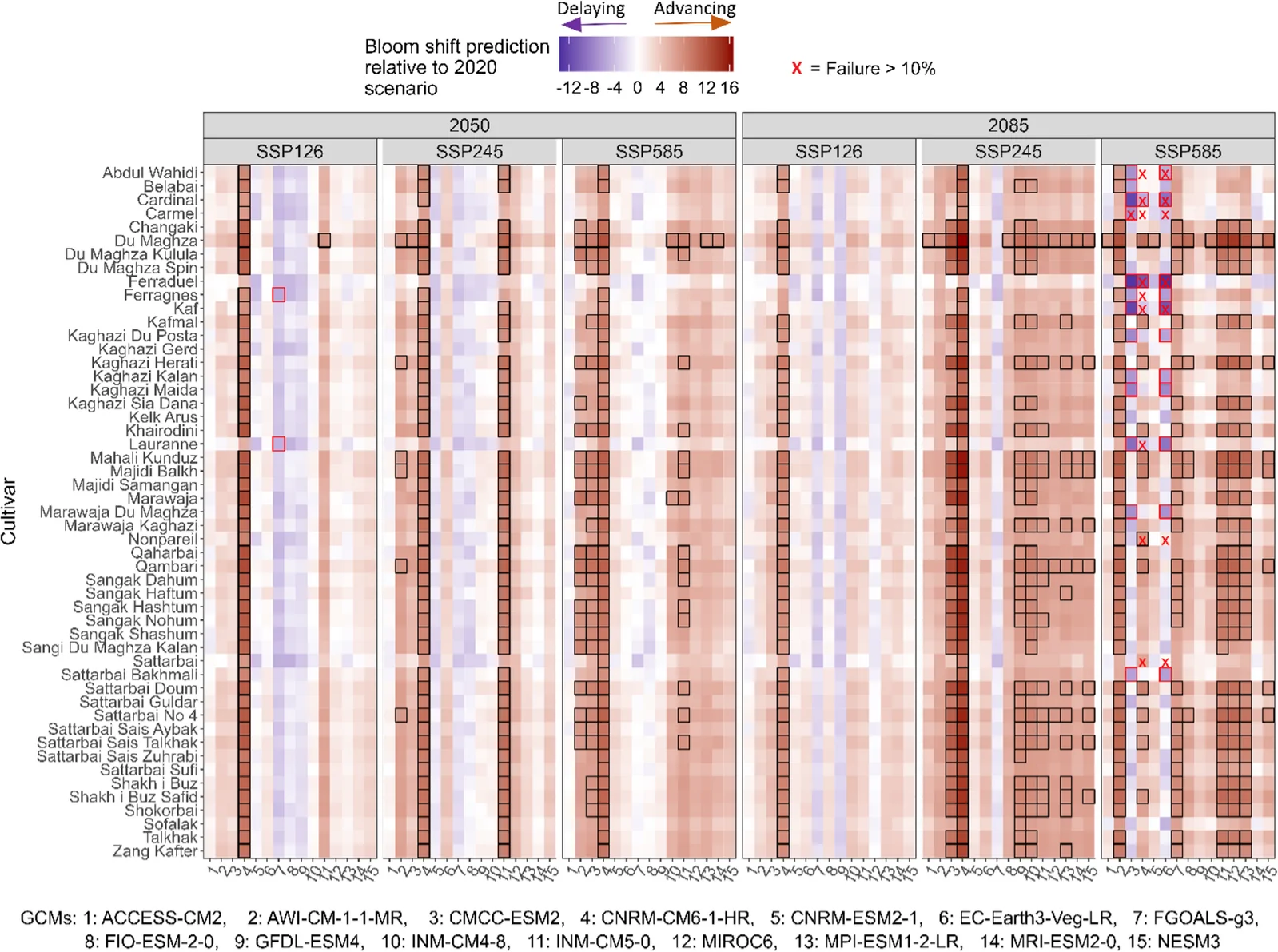
Predicted bloom dates for 51 almond cultivars under the future SSP scenarios. For each future SSP scenario (SSP1-2.6, SSP2-4.5, and SSP5-8.5), time point (2050 and 2085), and the 15 GCMs, the predicted bloom dates are calculated relative to the 2020 historical scenario for change prediction. Numbers from 1 to 15 on the x-axis represent the GCMs summarized in the plot. The red x-symbol represents a predicted bloom failure rate > 10%. Box symbols indicate strong changes in bloom dates; black boxes indicate a bloom advance of more than 7 days, whereas red boxes indicate a bloom delay of more than 5 days

Our analysis of future bloom date predictions revealed both advances and delays in flowering patterns across different almond cultivars (Fig. 4). Overall, cultivars exhibited bloom advances, with the maximum shift reaching up to 16 days. A small subset of cultivars showed delayed bloom, with the maximum delay being 12 days (Fig. 4). The extent of bloom advances increased as predictions transitioned from optimistic (SSP1-2.6) to intermediate (SSP2-4.5) and pessimistic (SSP5-8.5) scenarios for the 2050 and 2085 time points.

By 2050, across all cultivars, the shares of bloom advances and delays indicated 67% advances and 33% delays under the optimistic scenario, 72% advances and 28% delays under the intermediate scenario and 85% advances and 15% delays under the pessimistic scenario, compared to the 2020 baseline. Approximately 7% of predictions under the optimistic scenario, 13% under the intermediate scenario and 15% under the pessimistic scenario indicated bloom advances > 7 days. Bloom delays remained below 5 days across all SSP scenarios. However, by 2085, across all cultivars and SSP scenarios, the share of bloom advances indicated further increases, while bloom delays showed further decreases, except for the pessimistic scenario. The shares of bloom advances and delays stayed at 75% and 25% under the optimistic scenarios, 92% and 8% under the intermediate scenario and 82% and 19% under the pessimistic scenario. Likewise, the share of predictions indicating bloom advances > 7 days ranged from 6% under the optimistic scenario to 25% under both the intermediate and pessimistic scenarios. Bloom delays were generally small, staying below 5 days across all SSP scenarios and time points, except for a small subset of predictions (3%) for 2085 under the SSP5-8.5 pessimistic scenario, where delays exceeded 5 days.

We also observed considerable variation among the 15 GCMs in predicting future almond bloom dates. Among these models, ‘CNRM-CM6-1-HR’ consistently predicted the strongest bloom advances across cultivars, with a particularly pronounced effect on early-flowering cultivars, ranging from 8 to 16 days across all the scenarios and time points (Fig. 4). In contrast, for 2050, the models ‘FGOALS-g3’ and ‘FIO-ESM-2-0’ projected a general delay in flowering for all cultivars. By 2085, the models ‘CMCC-ESM2’ and ‘EC-Earth3-Veg-LR’ predicted the strongest delays in flowering, ranging from 5 to 12 days under the pessimistic SSP5-8.5 scenario (Fig. 4). In general, bloom delays were most prominent in late-flowering cultivars such as ‘Abdul Wahidi’, ‘Cardinal’, ‘Carmel’, ‘Ferragnes’, ‘Ferraduel’, and ‘Lauranne’. Conversely, bloom advances were most pronounced in several early- and intermediate-flowering cultivars.

We also observed a potential risk of bloom failure in many future projections. The likelihood of bloom failure was primarily predicted for the long term (2070–2100) under the pessimistic SSP5-8.5 scenario. For the near-term (2035–2065) scenario, no significant risk of bloom failure was found (Fig. 4). Notably, most of the bloom failure predictions were generated by GCMs that predicted relatively strong warming, including ‘CMCC-ESM2’, ‘CNRM-CM6-1-HR’, and ‘EC-Earth3-Veg-LR’.

## Discussion

The bloom prediction model showed high performance, with bloom predictions deviating from observed bloom dates, on average, by less than four days, according to the RMSEP. This is comparable to the accuracy of a PhenoFlex model for almonds in California, which showed RMSEP values between two and five days for calibration and validation datasets (Caspersen et al. 2024). However, the agro-climatic conditions in Kunduz are different from those in California. Kunduz has a semi-arid steppe climate, characterized by cold winters with snowfall and very hot summers (FAO and IIASA 2019). This underlines the importance of adjusting model parameters to reflect the local agro-climatic dynamics of Kunduz and to account for the specific chill and heat requirements of different cultivars. In doing so, we were able to characterize the chill and heat requirements of many indigenous cultivars—information that would otherwise only be obtainable through labor-intensive cutting-and-forcing experiments or long-term time series of phenology observations for statistical analyses (e.g. by Partial Least-Squares regression; Luedeling and Gassner 2012). The relatively short duration of our phenology dataset—spanning 12 years from 2011 to 2023, with a gap in 2013—presents some limitations, particularly the potential risk of overfitting when optimizing model parameters. However, this dataset represents the complete available record of almond phenology. In comparison, longer datasets have been used for similar studies elsewhere, such as the 60-year datasets (40 years for calibration, 20 years for validation) used for apple and pear phenology predictions in Germany (Luedeling et al. 2021) and almond bloom predictions in California (Caspersen et al. 2024). To overcome the constraints of a relatively short time series, we applied the combined-fitting approach of the PhenoFlex framework (Caspersen et al. 2026). By combining datasets across cultivars and refining them for local conditions, we were able to achieve high predictive performance despite limited temporal coverage.

Our results confirm that almond cultivars in Afghanistan are mostly early-flowering, supporting previous findings by Giordani et al. (2017), who reported the existence of only a few late-flowering varieties and also stated the problem of frost during flowering. We categorized all cultivars based on their bloom dates, which ranged from early to late flowering under the 2020 historical climatic scenario (Fig. 3). The difference between the earliest (February 21) and latest (March 15) flowering cultivars, at the 10% bloom stage, was approximately three weeks. In contrast, in Sfax, Tunisia, this range extends beyond one month, from January 8 to February 17 (Benmoussa et al. 2017). The shorter bloom duration indicates that early cultivars dominate the flowering period in Afghanistan. To avoid frost risk, breeding programs that cross early- and late-flowering cultivars might help achieve desirable traits, whereas considering adaptation measures would help growers manage the challenge of frost risk. The later bloom timing observed in Afghanistan compared to other regions highlights cross-regional climatic variation, which influences almond phenology.

Our study offers insights into the response of different almond cultivars to climatic conditions and the resulting bloom patterns. In particular, we observed cultivar-specific bloom advances and delays across future climate scenarios. These findings align with previous research on walnuts in California (Luedeling and Gassner 2012) and apricots in China (Guo et al. 2015), highlighting the contrasting effects of rising temperatures during the chilling and forcing phases. These earlier studies reported that the bloom delays experienced by some cultivars are related to increased temperature during the chilling phase, which causes a decrease in chill accumulation and ultimately delays spring phenology. We also observed bloom delays in some projections but these were not dominant at the cultivar level, likely due to the same underlying cause previously discussed. Such delays in flowering have recently been reported for almond cultivars in California (Caspersen et al. 2024). In contrast, rising temperatures during the forcing phase accelerate spring phenology and advance bloom dates (Guo et al. 2015). Accordingly, bloom advances were observed for most of the early-flowering Afghan almond cultivars (Fig. S4, Supplementary Information).

In addition to its regional and methodological significance, this study demonstrates the use of combined-fitting for a large germplasm dataset, enabling exploration of variability among cultivars and explaining its biological importance. As mentioned above, using this approach, we observed variation in bloom-shift magnitudes, including advances, delays, and stable responses (Fig. S4, S5 Supplementary Information). However, cultivars with bloom advances were more common overall because, in northern Afghanistan, the forcing effect generally outweighs the chilling effect for most cultivars in this dataset. Cultivars with low to moderate chill requirements showed strong advances, while those with high chill requirements exhibited weak or inconsistent responses (Fig. S4, Supplementary Information). For example, a local and early-flowering cultivar, ‘Mahali Kunduz’, has a chill requirement of 31.9 units and showed a median bloom advancement of 2 days under SSP1-2.6, six days under SSP2-4.5, and 6 days under SSP5-8.5 for 2050 and 2085 across all GCMs. Conversely, a foreign late-flowering cultivar, ‘Ferraduel’, with a chill requirement of 47.2 units, showed no bloom advancement under SSP1-2.6, a 1 day advancement under SSP2-4.5, and a 1 day delay under SSP5-8.5 (Fig. S4, Supplementary Information). This pattern also revealed that early-flowering cultivars experienced strong advances, whereas late-flowering cultivars remained comparatively stable (Fig. S5, Supplementary Information). In practice, this provides a foundation for applying this approach to other species with many cultivars but limited data, making it relevant for cultivar adaptation.

The PhenoFlex model also provides indications on potential bloom failure, revealing that several almond cultivars are vulnerable to the risk of bloom failure in the future. Notably, the failures were predicted for a smaller subset of predictions (2.2%) for 2085 under the pessimistic SSP5-8.5 scenario. The cultivars ‘Carmel’, ‘Nonpareil’, ‘Cardinal’, ‘Abdul Wahidi’, ‘Kaf’, ‘Ferraduel’ and ‘Sattarbai’ exhibited prominent failures mainly under the ‘CNRM-CM6-1-HR’ and ‘EC-Earth3-Veg-LR’ models. Similar risks have recently been reported for a range of temperate fruit trees, including almonds, in the Mediterranean region and central Europe (Caspersen et al. 2025). Both of these regions are projected to experience increased bloom failure risks under pessimistic long-term climate scenarios (2070–2100). This shows a shared vulnerability to climate change across regions and continents. The PhenoFlex model predicts bloom failure when agroclimatic requirements are not met. This is attributed to the underlying physiological mechanism whereby insufficient chill accumulation during warmer winters prevents the full release of endodormancy and leads to bloom failure (Campoy et al. 2011; Caspersen et al. 2025). Recently, a considerable risk of chill shortfall has been reported for Afghanistan, especially for warm parts of the country (Shinwari et al. 2025). These changes may have broader ecological and agricultural consequences, e.g. by affecting the abundance or scarcity of pollinators and pests and by causing asynchrony in flowering among cultivars, which may reduce pollination success. This is particularly important for almonds, most cultivars of which are self-incompatible and rely on cross-pollination. In Afghanistan, pollination is mostly accomplished by wild insects and wild bees, since beehives are rarely used systematically in the country’s orchards (Kaska et al. 2006). In the future, the projected changes may require identifying new combinations of cultivars that match in terms of bloom timing and extending the use of managed pollinators in orchards.

Our findings suggest that Kunduz province presents promising opportunities for investment in advanced commercial almond orchards. We did not observe a substantial risk of bloom shifts and chill losses in the near term (2035–2065), which likely applies to the neighboring almond-growing provinces as well. Our findings support the future projection by Masini and Giordani (2016) about perennial fruits and their potential expansion in terms of land investment (horizontal expansion), as in the near term, chill-related production challenges do not seem to be an alarming problem for fruit and nut growers (especially for almond orchards), particularly in the northern region. However, we acknowledge existing challenges in temperate fruit value chains, where orchard production systems are mostly traditional, with conventional practices, inconsistent genotypes within orchards and poor post-harvest handling (UC Davis and Roots of Peace 2010; Masini and Giordani 2016). Hence, it is crucial to enhance extension services for growers and other value chain actors to facilitate consistent production of high-quality almonds, improved pre- and post-harvest handling practices and updated market information.

## Conclusion

The observed changes in bloom timing, including advances, delays and failures, represent a clear deviation from historical patterns in response to climate change. The response of cultivars to climate change can be explained by the combination of chill and heat traits. However, these shifts in bloom timing may also present opportunities. Bloom shifts could help mitigate frost risk and disrupt the typical timing of insect and pest activity, potentially reducing damage to fruit crops. Moreover, producers might benefit from market advantages by supplying almonds earlier or later than usual. While late-flowering varieties naturally evade spring frost, most almond cultivars in Afghanistan are early-flowering, and our results indicate a further advancement in their flowering time. Therefore, it is essential to investigate regional spring frost events and implement protection measures to safeguard the production of almonds and other tree fruits and nuts.

## Supplementary Information

Below is the link to the electronic supplementary material.

Supplementary Material 1 (PDF 996 KB)

## Data Availability

The R code for data preparation, processing, analysis and generating figures for this study are available online in a public GitHub repository. (https://github.com/Atifshinwari/Almond-bloom-prediction-in-Afghanistan)
