# Supplementary material for: Climate change shifts almond bloom dates in Afghanistan
Source: Int J Biometeorol. 2026 Jul 28;70(8):224. doi: 10.1007/s00484-026-03288-0 (PMC13415282; doi:10.1007/s00484-026-03288-0)
Supplement: Supplementary file 1 — Supplementary Material 1 (PDF 996 KB) [file 484_2026_3288_MOESM1_ESM.pdf]

Supplementary information for "Climate change shifts almond bloom dates in Afghanistan"

Atifullah Shinwari[a], Lars Caspersen[a], Katja Schiffers[a], Eike Luedeling[a]

[a] Horticultural Sciences, Institute of Crop Science and Resource Conservation (INRES), University of Bonn, 53121 Bonn, Germany

Corresponding author: Atifullah Shinwari, atif92@uni-bonn.de

Performance of the calibrated model

In this study, we predicted the bloom dates of almond cultivars in Afghanistan, using the PhenoFlex framework. We first calibrated the framework using observed data and applied the resulting model to historical and future temperature data for bloom predictions. We applied this model to 51 almond cultivars in a combined-fitting approach. The overall performance of this model produced promising results, as indicated by two performance indicators: the Root Mean Squared Error of Prediction (RMSEP) and the Ratio of Performance to Inter-Quartile distance (RPIQ) (Fig. S1).

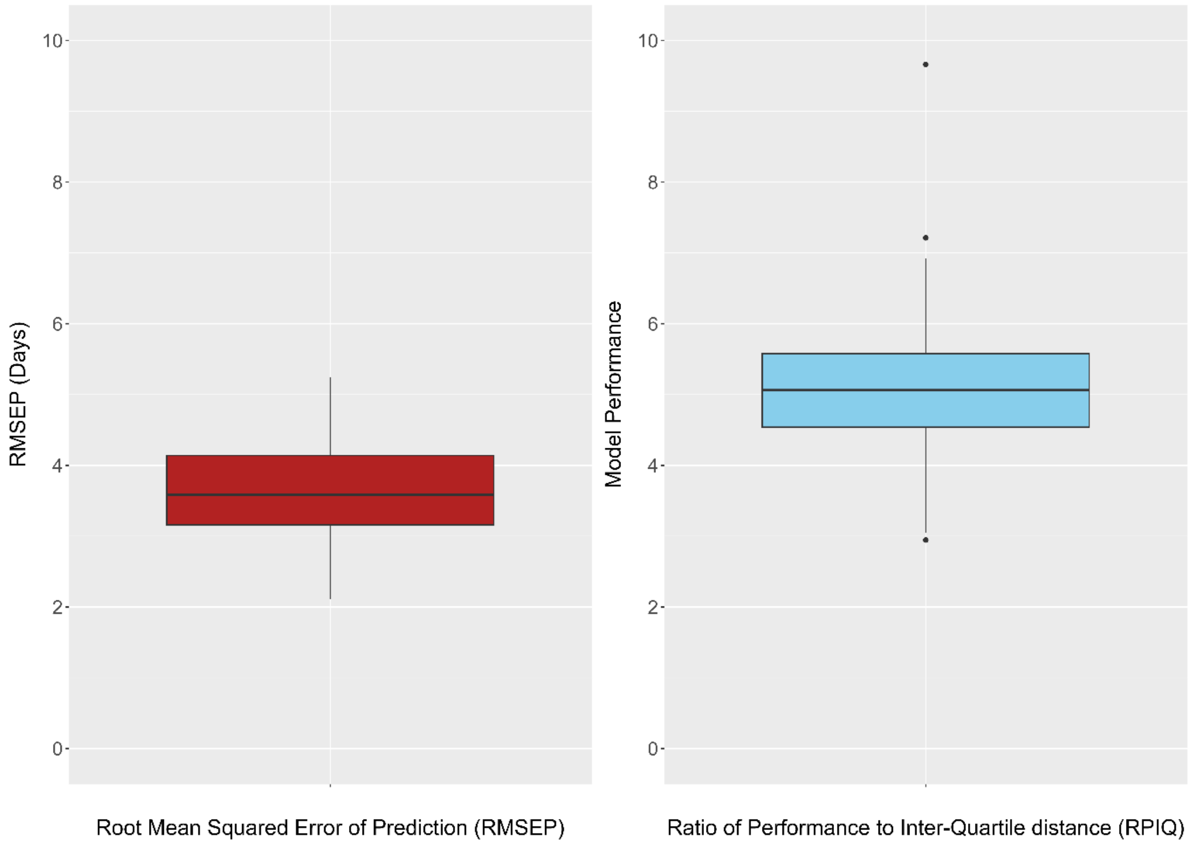

Fig. S1 Performance of the calibrated model for predicted and observed bloom dates of 51 almond cultivars, grown in the Kunduz province of Afghanistan.

Bloom predictions during model calibration

We performed the optimization process using a 10-fold cross-validation scheme, and we obtained bloom predictions for all 10 folds. These predictions are specific to the particular choice of calibration (75%) and validation (25%) dataset. We also calculated the RMSEP and RPIQ for predictions derived from individual folds (Fig. S2).

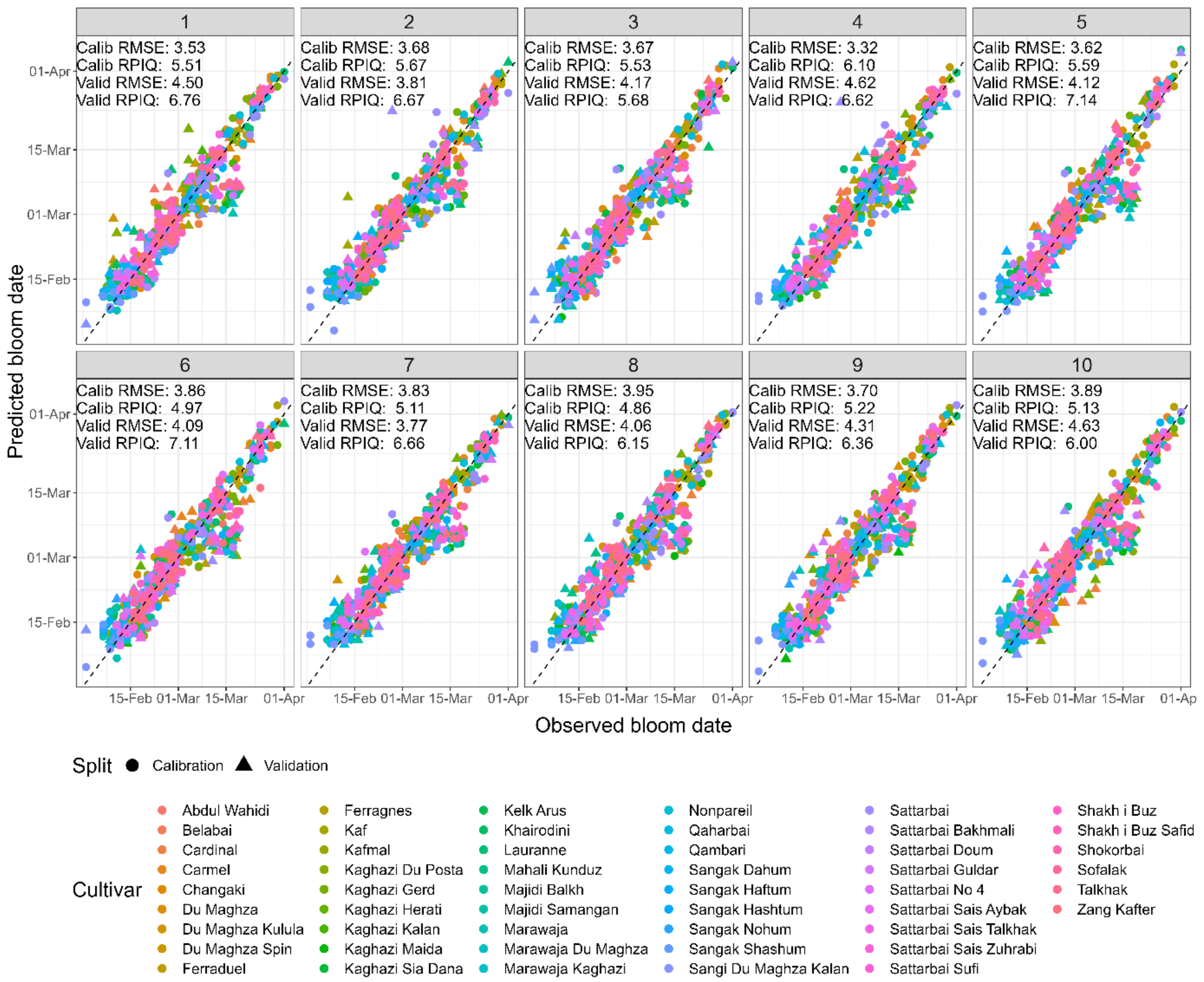

Fig. S2 Summary of the bloom date predictions for the calibration and validation datasets across the individual cross-validations for 51 almond cultivars.

Chill and heat accumulation relative to seasonal temperature

We analyzed the cultivar-specific chill and heat accumulation throughout the dormancy season, as predicted by the calibrated PhenoFlex model. Overall, the model showed high performance for all the cultivars. For instance, 'Belabai', an intermediate-flowering variety, is predicted to become sensitive to chill accumulation in early September and to heat accumulation in early December, in almost every year (Fig. S2). Bloom onset is triggered when sufficient chill and heat has accumulated. In 2014, the model predicted earlier bloom dates (green dashed line) than the observed bloom dates (solid green line). There was a rapid drop in temperature in February 2014, causing the heat responsiveness to remain constant. After a few days, the temperature experienced a sharp increase, thereby restoring heat responsiveness (Fig. S3).

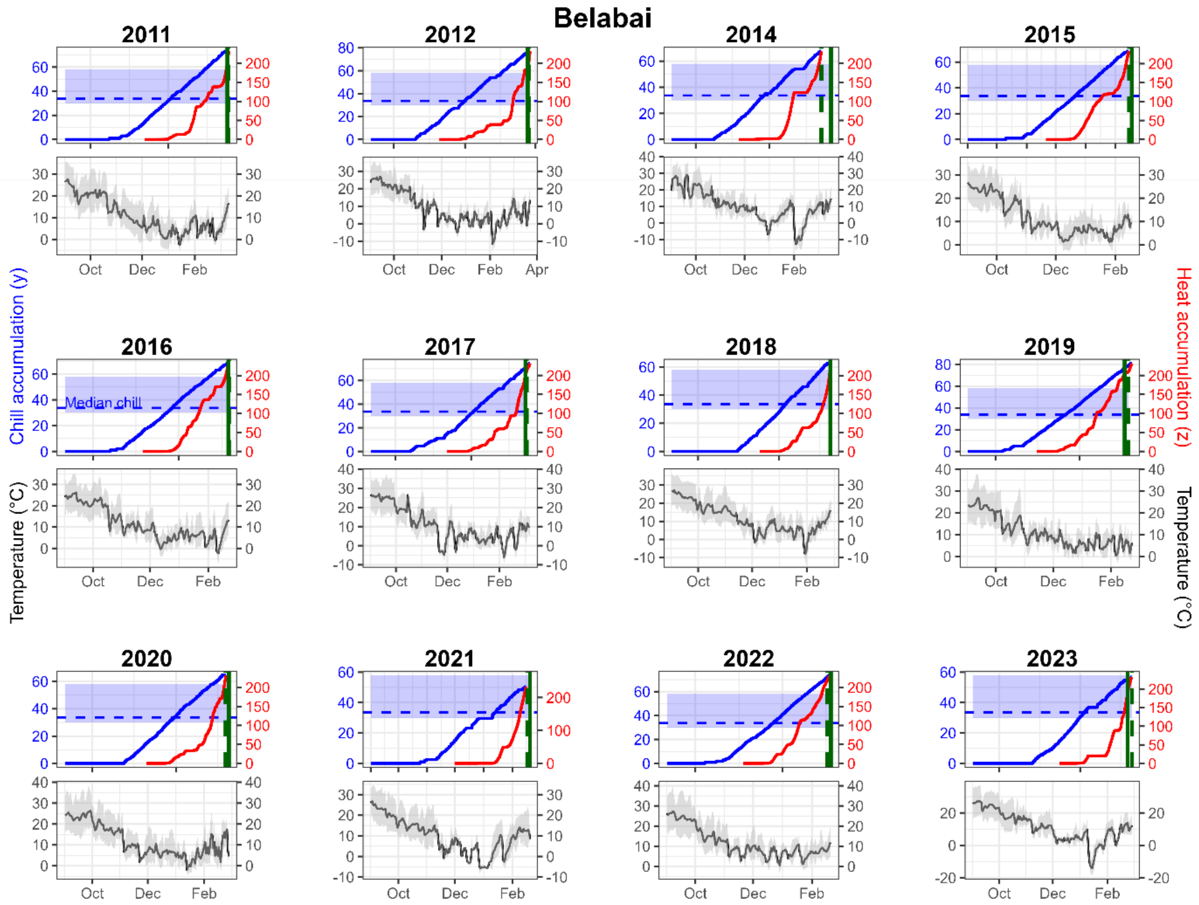

Fig. S3 Chill and heat accumulation in the calibrated PhenoFlex model using a combined-fitting approach. The blue line indicates chill accumulation, and the red line indicates heat accumulation. The solid green line represents the observed bloom date, whereas the dashed green line represents the predicted bloom date. The blue shaded area represents the upper and lower bounds of chill values (y_c) across the 10 cross-validation folds, and the blue dashed horizontal line represents its median value.

Chill and heat accumulation plots for all the cultivars are available in the GitHub repository accompanying this article (https://github.com/Atifshinwari/Almond-bloom-prediction-in-Afghanistan).

Projected cultivar bloom shift

We examined the extent of bloom shifts at the cultivar level for future SSP scenarios by using the median across two time points and all GCMs (Fig. S4). We grouped the cultivars into categories of strong (≥3.5 days), moderate (2-3.5 days), and weak (≤2 days) bloom-advancing based on median shift values. We assessed bloom shifts relative to the average chill requirements of each cultivar. We found a connection between the observed bloom advances and cultivar chill requirements, which were largely consistent across cultivars. A local cultivar, Du Maghza, exhibited the greatest bloom advances,

ranging from 3 days under SSP1-2.6 to 7 days under SSP2-4.6 and 8 days under SSP5-8.5. Bloom delays were rare; only 'Ferraduel' experienced a delay of 1 day under the SSP5-8.5 scenario (Fig. S4).

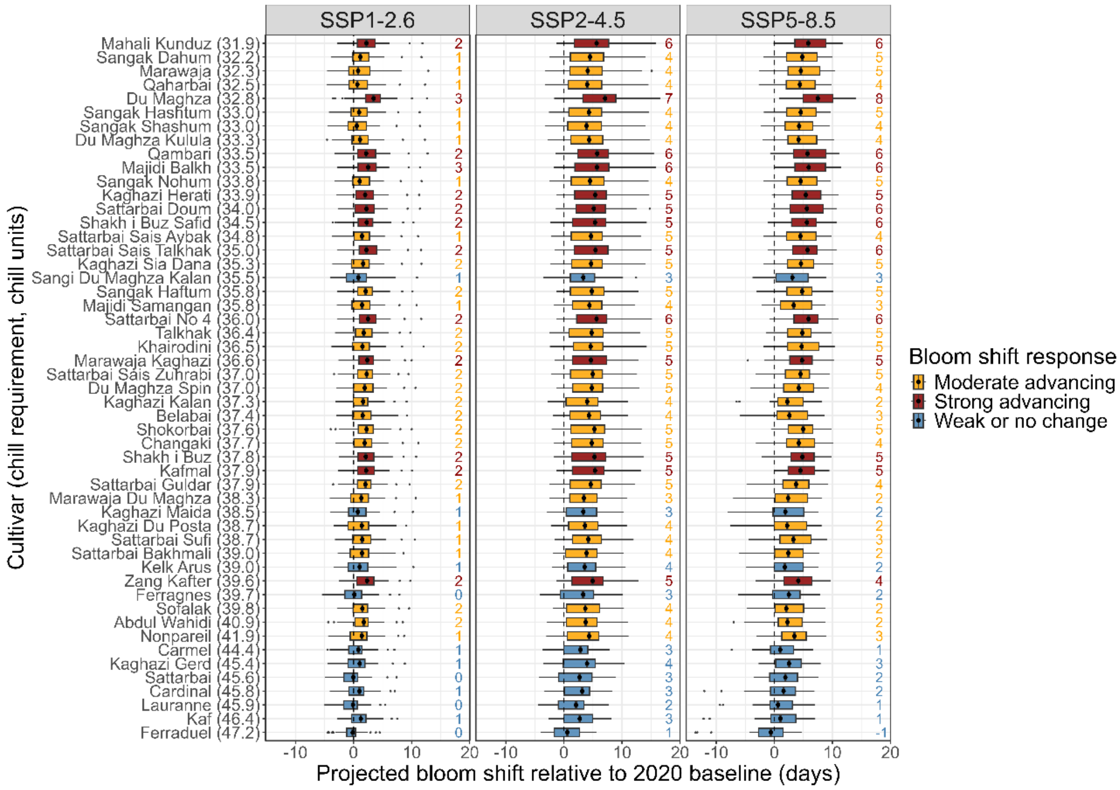

Fig. S4 Summary of the predicted future bloom shifts compared to the 2020 baseline scenario under the SSP scenarios. The values shown for each boxplot represent the median bloom shifts in days. The cultivars are ordered by mean chill units across all projections to illustrate how chill influences bloom patterns.

Furthermore, we analyzed the cultivars' flowering shifts and timing using a scatter plot to illustrate their distribution based on chill and heat needs (Fig. S5). We observed an overall smooth pattern from low- to high-chill cultivars. Cultivars with low chill (y_c) requirements show stronger advances, whereas those with high chill (y_c) requirements show little or no change in bloom timing. Similarly, early-flowering cultivars tend to have lower chill requirements and are more sensitive to warming; thus, they bloom earlier. Conversely, late-flowering cultivars cluster more around moderate chill and high heat requirements, resulting in little or no advance, as insufficient chill accumulation delays dormancy release.

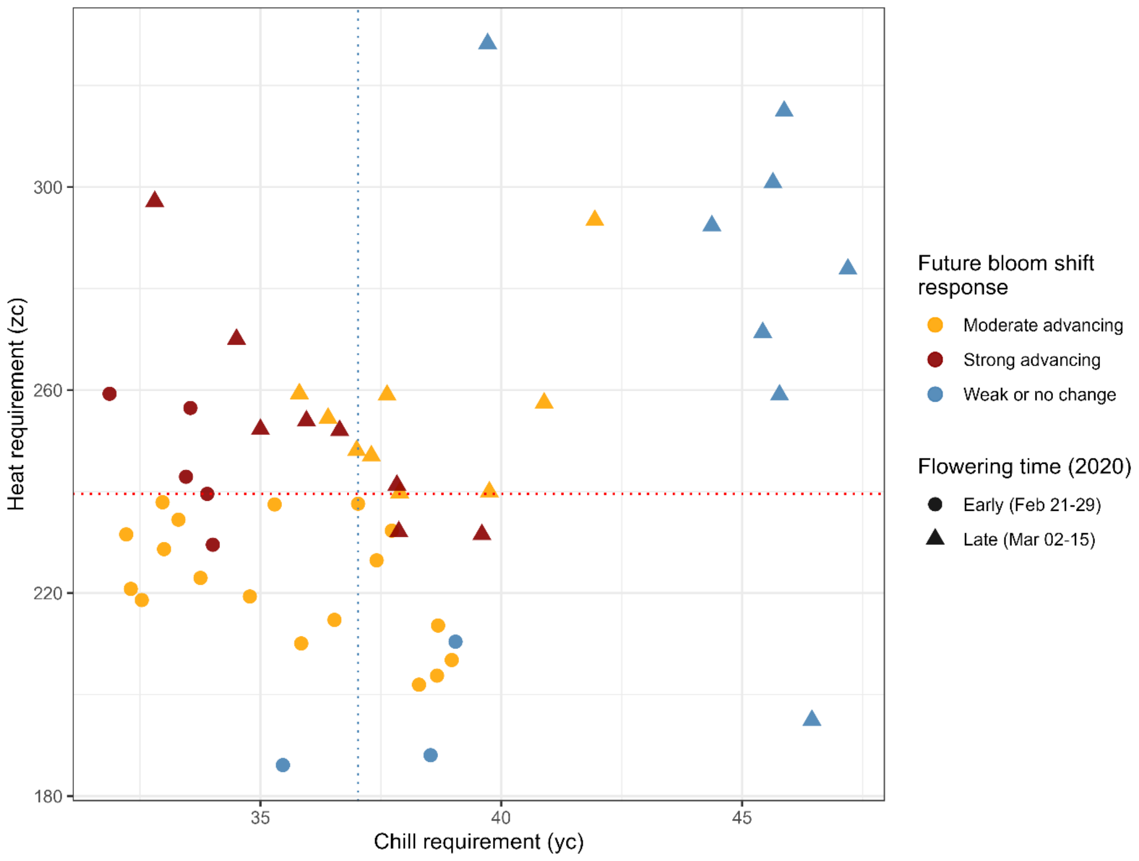

Fig. S5 Distribution of cultivars for future bloom shift responses and current flowering times based on chill and heat requirements. The blue dotted line indicates the median of chill requirements, and the red dotted line shows the median of heat requirements.
